# Case report: Optimized ruxolitinib-based therapy in an infant with familial hemophagocytic lymphohistiocytosis type 3

**DOI:** 10.3389/fimmu.2022.977463

**Published:** 2022-11-23

**Authors:** Daiki Niizato, Takeshi Isoda, Noriko Mitsuiki, Shuya Kaneko, Dan Tomomasa, Takahiro Kamiya, Masatoshi Takagi, Kohsuke Imai, Michiko Kajiwara, Masaki Shimizu, Tomohiro Morio, Hirokazu Kanegane

**Affiliations:** ^1^ Department of Pediatrics and Developmental Biology, Tokyo Medical and Dental University (TMDU), Tokyo, Japan; ^2^ Department of Clinical Research Center, Tokyo Medical and Dental University (TMDU), Tokyo, Japan; ^3^ Department of Community Pediatrics, Perinatal and Maternal Medicine, Tokyo Medical and Dental University (TMDU), Tokyo, Japan; ^4^ Department of Pediatrics, National Defense Medical College, Tokorozawa, Japan; ^5^ Center for Blood Transfusion and Cell Therapy, Tokyo Medical and Dental University Hospital, Tokyo, Japan; ^6^ Department of Child Health and Development, Tokyo Medical and Dental University (TMDU), Tokyo, Japan

**Keywords:** cytokine, familial hemophagocytic lymphohistiocytosis (FHL), HLH, hematopoietic cell transplantation (HCT), janus kinase, ruxolitinib

## Abstract

Familial hemophagocytic lymphohistiocytosis (FHL) is a rare and fatal autosomal recessive immune disorder characterized by uncontrolled activation of T and NK cells, macrophages, and overproduction of inflammatory cytokines. Early hematopoietic cell transplantation (HCT) is required for long-term survival. Current therapy is based on the HLH-94/2004 protocol, but is insufficient to fully control disease activity. This case report describes an infant with FHL type 3 who, despite initial therapy with dexamethasone and etoposide, showed aberrant cytokine levels, including interleukin-18 (IL-18), chemokine ligand 9 (CXCL9), soluble interleukin-2 receptor (sIL-2R), and soluble tumor necrosis factor receptor type II (sTNF-RII). The Janus kinase inhibitor ruxolitinib was therefore coadministered. The patient was treated with dose-adjusted ruxolitinib guided by cytokine profiles, and was successfully prepared for HCT. The results demonstrate the effectiveness and safety of dose-adjusted ruxolitinib as a bridging therapy for FHL, and the value of monitoring cytokine levels, especially IL-18, CXCL9, sIL-2R, and sTNF-RII, as disease-activity markers for FHL.

## Introduction

Familial hemophagocytic lymphohistiocytosis (FHL) is a fatal autosomal recessive immune disorder related to the dysfunction of cytotoxic, granule-mediated cell-death pathways. Prolonged activation of T and NK cells lacking the ability to kill target cells produces a high amount of cytokines, including interferon (IFN)-γ. This leads to the infiltration of activated macrophages and the production of interleukin (IL)-6, IL-18, and tumor necrosis factor (TNF)-α (1). Activation of T cells and macrophages plays a significant role in symptom development and organ damage in FHL. FHL type 3 (FHL3) is caused by a variant of the *UNC13D* gene encoding Munc13-4, which regulates the secretory process of cytotoxic granules of lymphocytes (2–4). In addition to the common clinical and laboratory findings, half of FHL3 patients present central nervous system (CNS) involvement and malignancy (4, 5). FHL is a fatal disease that requires early definitive diagnosis so that appropriate bridging therapy can be started until curative hematopoietic cell transplantation (HCT) is possible (4).

Immunomodulation of hemophagocytic lymphohistiocytosis (HLH) based on the pathophysiology and disease activity is warranted. However, specific cytokines that allow discrimination between FHL and secondary HLH have not been defined (6). Also, proteome analysis did not identify differential expression between primary and secondary HLH (7). Monitoring of serum cytokine profiles is effective in evaluating inflammatory status in cell therapies, infections, cancers, and auto-immune diseases (8). Time-course cytokine monitoring allows for appropriate selection and dosage of immunomodulators and is extremely important for understanding the pathogenesis of FHL. However, specific cytokines for monitoring disease activity have not been established in FHL patients.

FHL patients are predisposed to developing severe HLH even *in utero* or at birth, which can be fatal due to multiple organ failure, and often fail to survive until curative HCT (9–11). In the treatment strategy based on HLH-94/2004 protocols, HLH disease activity frequently recurs during steroid reduction and prolonged intervals of etoposide administration (12, 13). The HLH-2004 protocol has not improved pre-transplant patient mortality or improved survival (13), necessitating the need for novel therapeutic agents.

Ruxolitinib, a Janus kinase (JAK) inhibitor, has been used in the treatment of myelofibrosis, polycythemia vera, refractory acute and chronic graft-versus-host disease (GVHD), as well as COVID-19 acute respiratory distress syndrome and post-transplant idiopathic lung injury (14–19). Ruxolitinib is known to inhibit JAK1, JAK2, and signal transducer and activator of transcription (STAT) 3 pathways, thereby reducing downstream signaling and cytokine production, including IL-6 and IFN-γ (20–22). Reports of the use of ruxolitinib as an immunomodulator for FHL have been published (11, 23–29). However, the dose of ruxolitinib, combination of other therapies, and investigated cytokines were diversified.

In this case report, we describe an infant with FHL3 who underwent successful HCT following bridging therapy with ruxolitinib. The ruxolitinib dosage was adjusted based on monitored cytokine profiles.

## Results

### Case report

**Figure 1:**
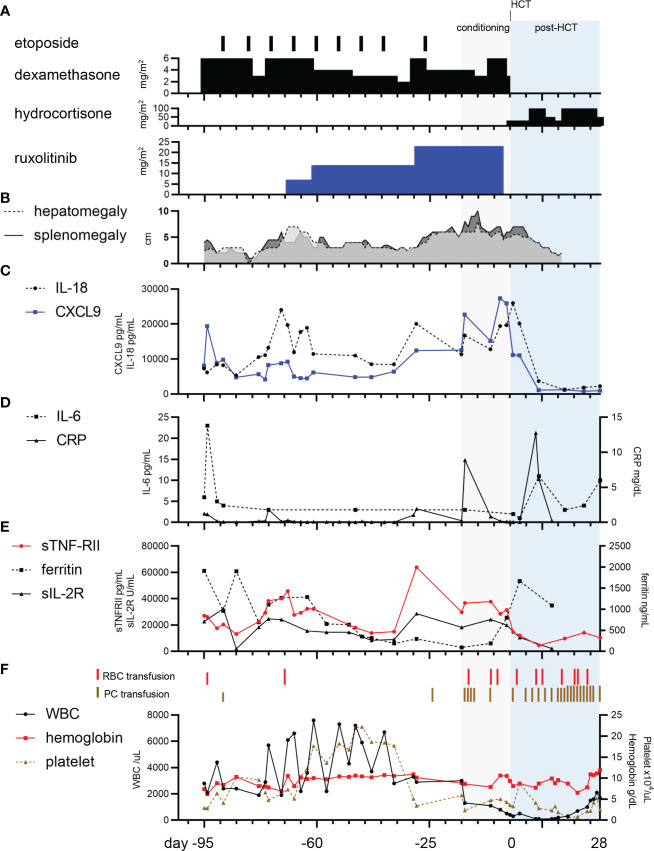
Clinical course for the patient. **(A)** Etoposide, dexamethasone, and ruxolitinib were used as bridging therapies. Hydrocortisone was given for maintenance therapy. **(B)** The clinical course of hepatosplenomegaly. **(C)** The panel shows the trajectory of chemokine ligand 9 (CXCL9) and interleukin-18 (IL-18). **(D)**. The panel shows the course of interleukin-6 (IL-6) and C-reactive protein (CRP). **(E)** The panel shows the course of ferritin, soluble interleukin-2 receptor (sIL-2R), and soluble tumor necrosis factor receptor type II (sTNF-RII) **(F)** The panel shows the timing of red blood cell (RBC) and platelet concentrate (PC) transfusion, and complete blood count tracking.

A 1-month-old boy was admitted to hospital with failure-to-thrive and fever. He was found to have thrombocytopenia (platelets 29 × 10^9^/L), hyperferritinemia (ferritin 1,912 ng/mL), and elevated soluble interleukin-2 receptor (sIL-2R) (26,489 U/mL), which suggested HLH (**Figures 1A, B**, **F**), and referred to our hospital. Upon admission, physical examination revealed fever, decreased oxygenation (SpO_2_ 93% on room air), hepatosplenomegaly, and a skin rash (**Figure 1B**). Complete blood count showed pancytopenia, with white blood cells 2,800/µL, neutrophils 840/µL, hemoglobin level 7.4 g/dL, and platelets 2.9 × 10^4^/µL (**Figure 1F**). Bone marrow aspiration at initial diagnosis provided insufficient material with peripheral blood contamination. Except for no confirmation of hemophagocytosis in the bone marrow aspiration, seven of the eight diagnostic criteria for HLH-2004 were fulfilled, namely fever, splenomegaly, pancytopenia, hypofibrinogenemia (1.09 g/L), absent natural killer (NK) cell activity, hyperferritinemia, and an increased sIL-2R level (30). Lumbar puncture, with cytology and cytokine evaluation, and brain magnetic resonance imaging showed no CNS involvement. On day one of admission (95 days before HCT), serum cytokine levels of IL-6, IL-18, chemokine ligand 9 (CXCL9), and soluble tumor necrosis factor receptor type II (sTNF-RII) were measured using ELISA according to the manufacturer’s instructions (IL-18: MBL, Nagoya, Japan; IL-6, CXCL9, and sTNF-RII: R&D Systems, Inc., Minneapolis, MN, USA) (31). All serum cytokine levels were elevated with IL-6 6 pg/mL (normal, <3 pg/mL), IL-18 7,300 pg/mL (normal, <500 pg/mL), CXCL9 8,116 pg/mL (normal, 31-83 pg/mL), and sTNF-RII 27,008 pg/mL (normal, 829-2,262 pg/mL) (**Figures 1C–E**). A CD107a assay showed no degranulation of NK cells, suggesting FHL. Flow cytometry showed decreased Munc13-4 expression. Sequencing analysis of the *UNC13D* gene, including in the parents, revealed the c.2346-2349del (p.Arg782fs) inherited from father, and tandem duplication of exons 7-12 inherited from mother, which were previously reported as pathogenic, leading to the definitive diagnosis of FHL3 (32, 33). Further details of the genetic diagnosis are described separately (34). An HLA-matched donor could not be found in the cord blood or bone marrow bank. Induction therapy with 6 mg/m^2^ dexamethasone (DEX) and 33 mg/m^2^ etoposide weekly was started based on a modified HLH-94 protocol. DEX administration was started at a two-thirds dose (**Figure 1A**). The dose of etoposide was determined by body weight using the following conversion formula in accordance with the AML-05 protocol for infants (35): Dose = 100 mg body surface area × (body weight (kg)/30). The dose was further reduced to two-thirds.

### The course of bridging therapy before conditioning for HCT

DEX was reduced from 6 to 3 mg/m^2^ 16 days after admission, which led to elevated levels of cytokines IL-18, CXCL9, sIL-2R, and sTNF-RII, while IL-6 remained below the cut-off level (**Figures 1C, D, E**). Despite DEX being increased to the initial dose on day 21, worsening of recurrent fever, hepatosplenomegaly, and elevated cytokine levels, indicated that the inflammation was difficult to control with the combination of DEX and etoposide (**Figures 1B, C, E**). Ruxolitinib was therefore initiated at 7 mg/m^2^ (2.5 mg/day) on day 27. After the ruxolitinib initiation, cytokine levels for IL-18, CXCL9, sTNF-RII, and sIL-2R showed a declining trend (**Figures 1C, E**). Instead of reduction of DEX from 6 mg/m^2^ to 4 mg/m^2^, the dose of ruxolitinib was increased from 7 to 14 mg/m^2^ on day 35, which lead to a gradual decrease of cytokine levels until day 60.

To delay HCT for the young infant and reduce complications, we attempted to extend the duration of etoposide administration and reduce the dosage of systemic steroids. However, the recurrent elevation of cytokine levels required preparation for early HCT. Thus, DEX was temporarily increased to 6 mg/m^2^ and ruxolitinib was increased to 23 mg/m^2^ on day 67 (29 days before HCT). We settled the timing of HCT when he reached at least 4-month-old, which is the youngest patient with FHL using posttransplant cyclophosphamide method (36).

### The course of HCT

The conditioning regimen, which included alemtuzumab 0.496 mg/kg, fludarabine 180 mg/m^2^, and busulfan AUC 65 mg×h/L, was started 15 days before HCT. Excellent outcomes for primary HLH have been reported with this reduced-intensity conditioning (37). Immediately after the administration of alemtuzumab, cytokine release syndrome (CRS), which includes fever, hypoxia, hepatomegaly with elevated hepatic enzymes, and rash, was observed. CRS was carefully managed with an additional dosage of the methylprednisolone, cytokine monitoring and laboratory tests. Ruxolitinib was discontinued one day before HCT because of possible negative effects on engraftment and a lack of sufficient data for continuation of ruxolitinib in GVHD prevention (38). Bone marrow transplantation from the haploidentical father (A-, B-, and C-mismatched) was performed when the patient was 4 months old. GVHD prophylaxis included posttransplant cyclophosphamide, tacrolimus, and mycophenolate mofetil (39). Cytokines IL-18, CXCL9, sIL-2R, and sTNF-RII immediately showed a profound reduction after HCT. Engraftment was achieved on day 25, complete chimerism was achieved on day 66. Although the patient developed sinusoidal obstructive syndrome (SOS), thrombotic microangiopathy (TMA) and pulmonary hypertension (PH), these complications were successfully managed. Ruxolitinib was used for TMA management after PH development during the course of haplo-HCT. Ruxolitinib was successfully used for GVHD prophylaxis instead of a calcineurin inhibitor and did not worsen the status of SOS, TMA, and PH. On day 286, the patient had remained without GVHD and recurrence of HLH, SOS, TMA, and PH after cessation of ruxolitinib and tacrolimus.

### Literature search

Among 38 papers found as part of the literature review of “ruxolitinib, hemophagocytic lymphohistiocytosis, FHL, and HLH” in PubMed, we selected 12 cases with primary HLH from case reports, retrospective, and prospective studies. The dose of ruxolitinib, combination of other therapies, and monitored cytokines were varied in 13 cases including our case. Except for one case without outcome record, 10 of 12 cases treated with ruxolitinib survived without any severe adverse events (**Table 1**).

**Table 1:** Literature review: Ruxolitinib for FHL in case reports and prospective study.

	Author (Year; ref)	Etiology of HLH	Age	Induction	Induction response	Ruxolitinib dose, duration, other therapies	Response after starting ruxolitinib	Cytokines used as biomarkers	HCT	Outcome
Case report	Marois et al. [2021 (24)]	*PRF1*	15 months	methylprednisolone 2mg/kg/day	partial clinical response	50mg/m^2^/day DEX 10mg/m^2^/day IT Continue 10 weeks	normalized neutropenia (48hr), disappearance of splenomegaly (10days)	NA	Yes	Alive
	Ramanan et al. [2020 (25)]	*UNC13D*	3 years	HLH-04	Recurrent with EBV infection	5 mg hydrocortisone 3 mg/kg/day continued 4 weeks	Clinical response	NA	Yes	Alive
	Yang et al. [2021 (11)]	*UNC13D*	6 days	HLH-94	Temporal clinical response	NA	Temporal clinical response	IFN-γ, IL-6, IL-10	No	Died (respiratory failure)
	**This report**	*UNC13D*	1 month	HLH-94 modified, 3 weeks to ruxolitinib	Temporal clinical response	2.5mg - 8mg (7 - 23 mg/m^2^)	Temporal clinical response	CXCL9, IL-6, IL-18, sTNF-RII	Yes	Alive
	Zhao et al. [2020 (23)]	*RAB27A*	14 years	HLH-04	deteriorated after 6 weeks	started 10mg, and increased to 50mg/day after primary graft failure with TMA of salvage haploidentical allo-SCT	Clinical response	IFN-γ, IL-10	Yes	Alive
	Zhang et al [2021 (29)]	*RAB27A*	3 years	HLH94 with ruxolitinib	NA	HLH94 with ruxolitinib 10mg/day	Clinical response	IFN-γ	Yes	Alive
Retrospective study (9 patients)	Wei et al. [2020 (27)]	*PRF1* *PRF1*	2 years 6 month 5 years	HLH-94, for 3 weeks to ruxolitinib	Recurrent Refractory	<10 kg, 5mg/day 10-25 kg, 10mg/day ≥25 kg, 20 mg/day	PR after 1 week, maintain CR >10 months	IFN-γ, IL-6, IL-10	NA	Alive Alive
Retrospective study (34 patients)	Wang et al. [2020 (26)]	*PRF1*	NA	HLH-94 based, > 2 weeks	NA	<14 years, <25 kg, 5mg/day <14 years, ≥25 kg, 10mg/day ≥14 years, 20 mg/day glucocorticoid ± hydroxychloroquine sulfate	NA	IFN-γ, IL-18, MIP-1α, IP-10 improved in 25 cases with PR or CR	NA	NA
Prospective study (52 patients)	Zhang et al. [2022 (28)]	*PRF1* *UNC13D* *UNC13D* *XIAP*	2.3 years 1 month 5.1 years 3.7 years	NA	NA	≤10 kg, 5mg/day 10 < - ≤ 20 kg, 10mg/day >20 kg, 20 mg/day	CR 3 of 4	IFN-γ	NA	Alive 3 of 4

CR, complete response; CXCR9, chemokine ligand 9; DEX, dexamethasone; EBV, Epstein-Barr virus; HCT, hematopoietic cell transplantation; HLH, hemophagocytic lymphohistiocytosis; IFN-γ, interferon-γ; IL. Interleukin; IP-10, interferon-γ-inducible protein; IT, intrathecal; MIP-1α, macrophage inflammatory protein; NA, not available; OR, overall response rate; PR, partial response; TMA, thrombotic microangiopathy.

## Discussion

In this case study, the dose adjustment of ruxolitinib, guided by cytokine monitoring, was an effective bridging therapy, and minimized the required dose of etoposide and DEX. This strategy also contributed to the decision of HCT timing with maintaining performance status. However, the cytokine levels of IL-18, CXCL9, sIL-2R, and sTNF-RII remained elevated above cutoff levels, suggesting that adequate doses of ruxolitinib combined with HLH94/2004-based therapy would be necessary for the treatment of FHL.

Past reports showed that cytokine levels, including IL-6 (cutoff level, 37.25 pg/mL), at initial diagnosis predict early death in children with HLH (40). The IL-6 level in our case was consistently below this cutoff (**Figure 1D**). The CXCL9/IL-6 ratio is discriminately elevated in HLH compared with systemic inflammatory response syndrome/sepsis (7). Similarly, we observed that serum CXCL9 which is IFN-γ-inducible CXCR3 ligands and reflecting amplification of IFN-γ signaling wathway (41), was consistently higher than the normal range throughout the clinical course in our case (**Figures 1C, D**). IL-18, sTNF-RII, and sIL-2R showed a similar trend to CXCL9 (**Figures 1C, E**). During the disease course, the patient had three episodes of fever without any apparent sign of infection, negativity in blood cultures, and the highest CRP level was 1.9 mg/dL. Thus, we monitored cytokines, which directly reflected FHL activity in our case. Further validation is warranted to determine whether these cytokine profiles are effective to monitor disease activities of FHL.

Effective salvage therapy has not been established for refractory FHL. A retrospective study using rabbit antithymocyte globulin (ATG) showed that one-third of patients relapsed after first course of ATG, and 19 of 38 patients who received HCT after first or second-line ATG have been cured. The median time between ATG therapy and HCT was 6 weeks (42). Alemtuzumab as salvage therapy showed partial responses and response was limited to two weeks (43). Both are not suitable salvage therapies for 1-3 months old infants who are required to wait for sufficient growth before HCT. Infectious complications and cytokine release syndrome are concerns on both therapies. Thus, alternative therapies are urgently required.

Hyperactivation of effector CD8+ T cells with overproduction of IFN-γ contributes to FHL pathogenesis (44). In a mouse model of FHL2, loss of IFN-γ receptors in hepatocytes reduced liver dysfunction, suggesting that a non-hematopoietic response is also crucial in developing FHL hepatitis (45). These findings suggest that targeting IFN-γ may be a promising treatment strategy. However, a recent clinical trial using the anti-IFN-γ monoclonal antibody emapalumab has not been successful (46). Despite the combination of emapalumab and dexamethasone, only 14 out of 34 cases (41%) survived without additional therapy. In addition, only 70% of patients in the emapalumab-treated group bridged to HCT, which was inferior to the 80% in the HLH-2004 study (13, 46, 47).

Ruxolitinib inhibits the JAK/STAT pathway, which is one of the main cytokine signaling pathways in T-cell activation and the maintenance of activation (22). The mouse FHL2 model suggested that ruxolitinib inhibits HLH through IFN-γ-dependent and independent pathways (48). Ruxolitinib acts not only on T cells, but also on human macrophages and inhibits the production of both IFN-γ and IL-6 (49). Our literature review showed that ruxolitinib was used for 13 FHL patients, including our case (**Table 1**). In a recent single-arm study involving four FHL patients, three of them responded to ruxolitinib monotherapy. The study investigated 52 cases, including systemic autoinflammatory-associated HLH, chronic active Epstein-Barr virus infection (CAEBV), and EBV-HLH. The cytokine profiles, including sIL-2R, ferritin, and IFN-γ, of good responders normalized within two weeks (28), whereas non-responders required additional therapies. In the report, cytokine profiles of four patients with FHL were not shown (28). Cytokine profiles in our case and neonatal-onset FHL showed a non-responder pattern, suggesting that additional therapies are required for these cases (11). Except for one case without outcome information, 10 out of 12 cases were successfully managed by additional ruxolitinib administration (**Table 1**). In recent a case of Griscelli syndrome type 2, ruxolitinib was used with HLH-94-based therapy for initial induction, and successfully led to HCT (29). Emapalumab and ruxolitinib combination therapy for a refractory CAEBV case has been reported (50). However, the dose of ruxolitinib, combination of other therapies, and investigated laboratory markers, including cytokines, were varied. The prospective clinical trial (NCT04551131) is crucial for determining response using 50mg/m^2^/day of ruxolitinib with dexamethasone for FHL. Selection of inflammatory markers and determining the appropriate cutoff value will be required in prospective studies.

## Concluding remark

Cytokine monitoring and trends of IL-18, CXCL9, sIL-2R, and sTNF-RII might reflect disease activity in FHL. Monitoring cytokine levels and administering ruxolitinib, combined with the HLH-94/2004 protocol or other recent target therapies, may be a potent bridging therapy for FHL patients.

## Data availability statement

The datasets for this article are not publicly available due to concerns regarding participant/patient anonymity. Requests to access the datasets should be directed to the corresponding author.

## Ethics statement

The studies involving human participants were reviewed and approved by the ethics boards of the Tokyo Medical and Dental University (TMDU) (G2019-004). Written informed consent to participate in this study was provided by the participants’ legal guardian/next of kin.

## Author contributions

DN and TI wrote the manuscript. Literature review: DN and TI. Measurement of cytokines: SK and MS. Acquisition and interpretation of data: DN, TI, NM, SK, DT, TK, MS, and HK. Supervision of the study: MT, KI, MK, MS, and TM. Conceptualization of the study and editing the manuscript: HK. All authors contributed to the article and approved the submitted version.

## Funding

This work was supported in part by JSPS KAKENHI Grant Number (21H02878) and Takeda Science Foundation to TI and by the joint research grant with Medical and Biological Laboratories Co., Ltd (MBL) to MS. KAKENHI contributed an English editing fee. KAKENHI and Takeda Science Foundation contributed open access publication fee. The Joint research grant with MBL covered the reagents for cytokine monitoring. The funder, Medical and Biological Laboratories Co., Ltd, was not involved in the study design, collection, analysis, interpretation of data, the writing of this article, or the decision to submit it for publication.

## Acknowledgments

We are grateful to Drs. Takayuki Miyamoto and Takahiro Yasumi for performing flow cytometry of Munc13-4. We thank the patient and his parents for participating in this study. We also thank the staff at the Department of Pediatrics, Tokyo Medical and Dental University. We thank Barbara Spathelf, PhD, from Edanz (https://jp.edanz.com/ac) for editing a draft of this manuscript.

## Conflict of interest

The authors declare that the research was conducted in the absence of any commercial or financial relationships that could be construed as a potential conflict of interest.

## Publisher’s note

All claims expressed in this article are solely those of the authors and do not necessarily represent those of their affiliated organizations, or those of the publisher, the editors and the reviewers. Any product that may be evaluated in this article, or claim that may be made by its manufacturer, is not guaranteed or endorsed by the publisher.
